# Spontaneous splenic rupture, mesenteric ischemia and spinal infarction after aortic repair for acute type A dissection in a patient with sickle cell trait

**DOI:** 10.1007/s11748-020-01520-1

**Published:** 2020-10-22

**Authors:** Makoto Toyoda, Tadashi Kitamura, Kouki Nakashima, Yoshikiyo Matsunaga, Masaki Nie, Kagami Miyaji

**Affiliations:** 1 Department of Cardiovascular Surgery, Ebina General Hospital, 1320 Kawaraguchi, Ebina, Kanagawa 243-0433 Japan; 2 Department of Cardiovascular Surgery, Kitasato University School of Medicine, Minami-ku Kitasato 1-15-1, Sagamihara, Kanagawa 252-0374 Japan

**Keywords:** Aortic dissection, Mesenteric ischemia, Sickle cell trait, Splenic rupture, Spinal infarction

## Abstract

Sickle cell trait (SCT), a benign hematological condition affecting approximately 300 million individuals globally, is associated with an increased risk of vaso-occlusive disease. However, the risks related to surgery employing cardiopulmonary bypass in patients with SCT are not well established. Herein, we report the case of a 27-year-old African American man with SCT who underwent an emergency aortic repair for acute Stanford type A aortic dissection using hypothermic circulatory arrest. The patient developed a sickle cell crisis, which was followed by spontaneous splenic infarction and rupture, nonocclusive mesenteric ischemia, and spinal infarction.

## Introduction

Sickle cell trait (SCT) is a benign condition resulting from a heterozygous mutation of beta-globin gene allele that encodes hemoglobins (Hbs) A and S. Typically, individuals with SCT have no symptoms; however, they are at a higher risk of venous thromboembolism, chronic kidney disease and exertional rhabdomyolysis [1]. Approximately 8% of African Americans and approximately 300 million individuals globally have SCT [2]. However, few studies to date have reported on patients with SCT who have undergone open heart surgery using cardiopulmonary bypass (CPB), during which hypothermia, hypoxemia, or acidosis can result in vaso-occlusive disease. Here, we report the case of a patient with SCT who presented with acute Stanford type A aortic dissection and developed splenic rupture, nonocclusive mesenteric ischemia, and spinal infarction following an emergency aortic repair.

## Case

**Fig. 1:**
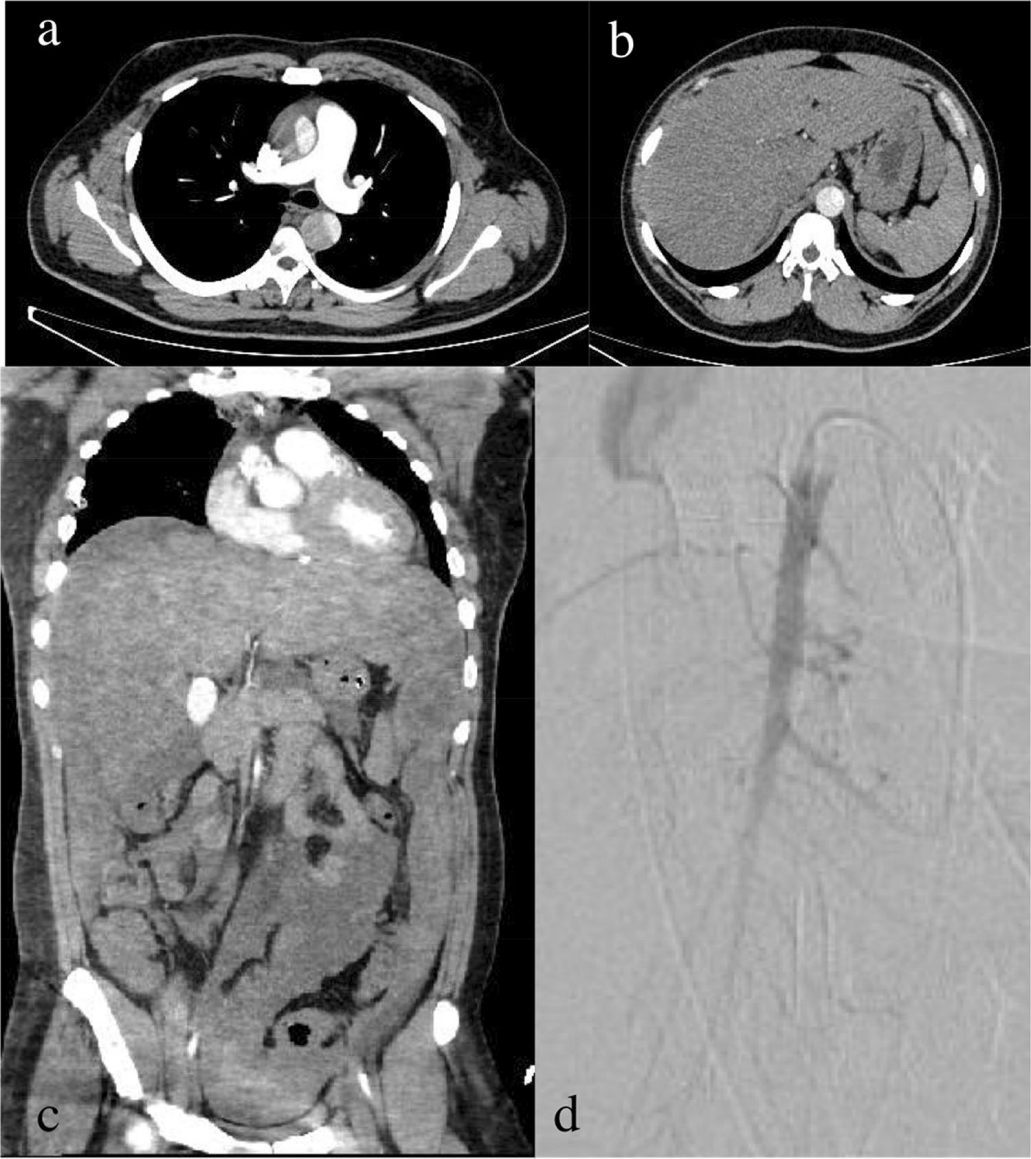
**a**, **b** Contrast-enhanced computed tomography (CT) showing type A aortic dissection and splenomegaly. **c** CT after the first operation showing ischemic small intestine showing a reduced contrast effect in peripheral region of superior mesenteric artery (SMA) and maintained contrast effect in the main trunk of SMA, along with the spleen and the liver infarct. **d** Angiography image showing reduced vessel filling in the peripheral region of the superior mesenteric artery

A 27-year-old African American man with untreated hypertension presented with precordial pain. He had previously been diagnosed with SCT based on a sickle hemoglobin level of 40.4% during a screening test while newly employed. He had never experienced complications correlated with SCT. His blood pressure was 142/74 mm Hg, and his heart and respiratory rates were 54/min and 20/min, respectively. Blood oxygen saturation and body temperature were normal. Laboratory data were normal except for an increase in D-dimer (6.0 μg/mL) and C-reactive protein (2.7 mg/dL). Contrast-enhanced computed tomography (CT) showed Stanford type A aortic dissection and splenomegaly measuring 13 cm in the maximum length without malperfusion. (Fig. 1a, b). The patient was transferred to the operating theater for emergency aortic repair.

Following median sternotomy, CPB was initiated with direct aortic true lumen cannulation and right atrial cannulation. Circulatory arrest with selective cerebral perfusion was initiated at a bladder temperature of 30 °C, and the ascending aorta was replaced. The operation, cardiopulmonary bypass, and circulatory arrest times were 6 h and 2 min, 2 h and 34 min, and 43 min, respectively. The amount of bleeding was 605 mL. 1400 mL of red cell concentrates, 2520 mL of flesh frozen plasma and 250 mL of platelet concentrate were transfused. Acidosis and hypoxemia after starting CPB were not well controlled (pH 7.26–7.29, pO_2_ 76.6–86.3). The operation was completed without apparent complications.

**Fig. 2:**
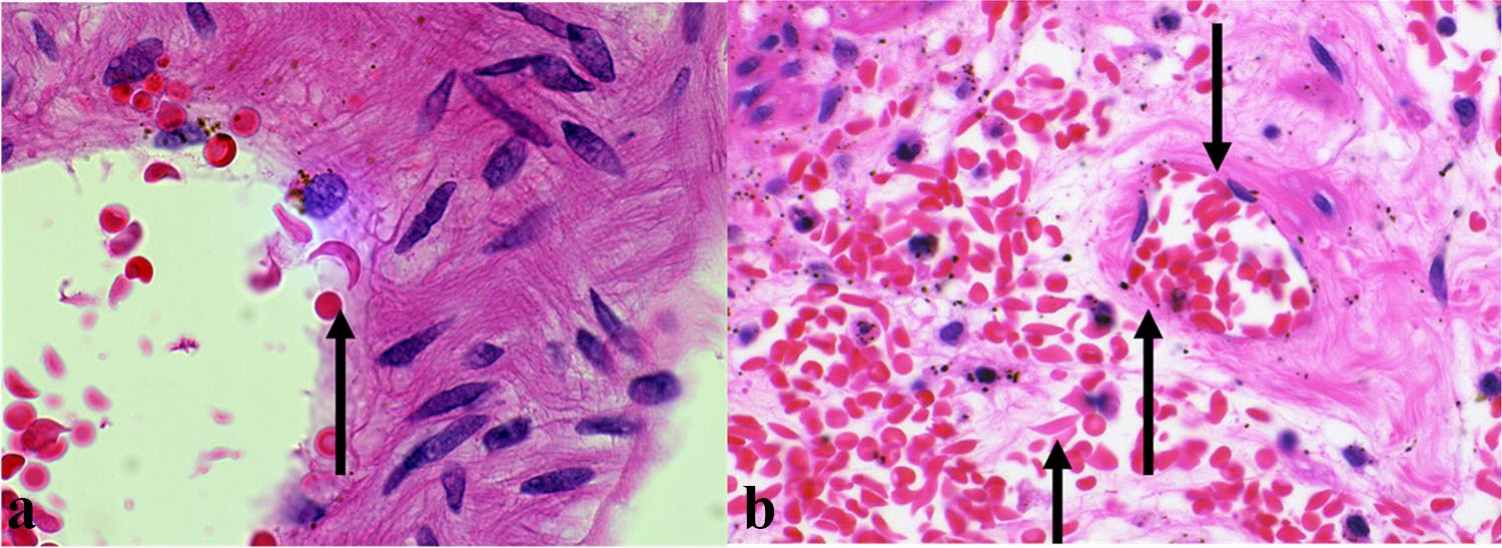
**a** Adhesion of a sickle cell to the ileum vessel. **b** Arterioles and sinusoids in the spleen filled with sickle cells

After the surgery, the patient experienced persistent bleeding, which required re-exploration. Laboratory examinations showed liver and renal dysfunction (aspartate aminotransferase, 2141 U/L; alanine aminotransferase, 815 U/L; lactate dehydrogenase, 3570 U/L; creatinine, 1.65 mg/dL) with significant coagulopathy (platelet count, 32,000/µL; fibrinogen, 91 mg/dL; prothrombin time-international normalized ratio, 1.99; activated partial thromboplastin time, 46.3 s), suggesting disseminated intravascular coagulation (DIC). Three hours later, the patient developed abdominal distension with profound shock and a blood pressure of 55/38 mm Hg, acidosis with an increase in lactate (9.1 mmol/L) and anemia (Hb 5.3 g/dL). CT showed a reduced contrast effect in the peripheral branch of the superior mesenteric artery, ischemic small bowel, and liver and spleen infarct (Fig. 1c). Angiography showed spasm and reduced vessel filling of the superior mesenteric artery (Fig. 1d). Emergency laparotomy revealed a large tear with active bleeding in the spleen and necrosis of the segmental ileum. Consequently, splenectomy and ileal resection were performed. The total amount of bleeding after the first surgery was 8500 mL. 2520 mL of red cell concentrates and 2160 mL of flesh frozen plasma were transfused during the laparotomy operation. On histopathological examination, the spleen was enlarged (180 × 95 × 50 mm) and had hemorrhagic necrosis. The resected ileum also showed ischemic alteration and necrosis. Sickle cells adhered to the endothelium and aggregated with each other (Fig. 2a, b). The next day, he was diagnosed with sepsis with peritonitis, kidney, and liver failure. Treatment with antibiotics, continuous hemodiafiltration, and steroid therapy was initiated. Seven days later, when the patient recovered from the anesthesia, he had developed paraplegia. Spinal magnetic resonance imaging showed diffuse subacute spinal infarction with hemorrhage. The patient was transferred to another hospital 31 days after the first operation.

## Discussion

With the increase in globalization, surgeons in Japan must sometimes perform emergency surgery on non-Japanese patients who have race-specific and unfamiliar diseases, including SCT. Acute type A aortic dissection typically requires emergency aortic repair with CPB and hypothermic circulatory arrest. Conversely, hypothermia, hypoxemia or acidosis induced by CPB can result in serious vaso-occlusive disease in patients with SCT [3, 4]. In our case, the patient developed multiple organ failure consistent with sickle cell crisis following the aortic repair. Sickle cell crisis is an acute complication of sickle cell disease (SCD) resulting from red cell sickling, and it presents in a variety of pathologies, such as acute chest syndrome, stroke, priapism, hepatobiliary complications, splenic sequestration, and acute renal failure [5]. The red cells of patients with SCD have a sickle-like appearance because of HbS polymerization, which is inhibited by HbA in patients with SCT, i.e., heterozygotes [6]. However, in SCT, red cell deoxygenation and low pH shortens the time taken for HbS polymerization and consequently enhances red cell adhesion to the endothelium and heterocellular aggregate formation. This results in a prolonged erythrocyte microvascular transit time, subsequently leading to vaso-occlusion and organ failure in patients with SCD [7]. In the present case, other etiologies like hemorrhagic shock and malperfusion syndrome could be considered for multiple organ failure; however, histopathological findings revealed arterioles and sinusoids filled with sickle cells and strongly indicated sickle cell crisis. We concluded that the multiple organ complications most likely resulted from sickle cell crisis and that the other factors possibly exacerbated severe organ failure. To date, no study has investigated aortic dissection in patients with SCT. Few published studies have mentioned the association between SCD and surgery using CPB. However, the measure of intraoperative management has not yet been established [8]. Further studies are necessary to elucidate the risk factors associated with SCT in aortic dissection. Although African countries have high number of patients with SCT [9], as globalization progresses, we should update the operative information for patients with SCT, particularly in Asian countries, where there are very few of them.

## Conclusion

We reported a patient with SCT who underwent emergency aortic repair for acute type A aortic dissection and developed spontaneous splenic rupture, mesenteric ischemia, and spinal infarction associated with sickle cell crisis. The utmost attention should be given to preventing sickling in patients with SCT who undergo surgery using CPB and hypothermia.

## References

[1] Naik RP, Smith-Whitley K, Hassell KL, Umeh NI, de Montalembert M, Sahota P (2018). Clinical outcomes associated with sickle cell trait. Ann Intern Med.

[2] Grant AM, Parker CS, Jordan LB, Hulihan MM, Creary MS, Lloyd-Puryear MA (2011). Public health implications of sickle cell trait: a report of the CDC meeting. Am J Prev Med.

[3] Yousafzai SM, Ugurlucan M, Al Radhwan OA, Al Otaibi AL, Canver CC (2010). Open heart surgery in patients with sickle cell haemoglobinopathy. Circulation.

[4] Crawford TC, Carter MV, Patel RK, Suarez-Pierre A, Lin SZ, Magruder JT (2017). Management of sickle cell disease in patients undergoing cardiac surgery. J Card Surg.

[5] Yawn BP, Buchanan GR, Afenyi-Annan AN, Ballas SK, Hassell KL, James AH (2014). Management of sickle cell disease summary of the 2014 evidence-based report by expert panel members. JAMA.

[6] Kumar V, Abbas AK, Aster JC, Kumar V (2018). Hematopoietic and lymphoid system. Robbins basic pathology.

[7] Stuart MJ, Nagel RL (2004). Sickle cell disease. Lancet.

[8] Frank E, Ernest A, Mark T, Martin T, Kow EM, Ernest OA (2014). Hypothermic cardiopulmonary bypass without exchange transfusion in sickle-cell patients: a matched-pair analysis. Interact Cardiovasc Thorac Surg.

[9] Twaito R (2016). Sickle cell trait: a benign state?. Acta Haematol.

